# Cytogenetic description of the Amazonian brown brocket *Mazama nemorivaga* (Artiodactyla, Cervidae)

**DOI:** 10.3897/CompCytogen.v7i1.4314

**Published:** 2013-03-18

**Authors:** Bruno Ferreto Fiorillo, Javier Adolfo Sarria-Perea, Vanessa Veltrini Abril, José Maurício Barbanti Duarte

**Affiliations:** 1 Núcleo de Pesquisa e Conservação de Cervídeos (NUPECCE), Departamento de Zootecnia, Faculdade de Ciências Agrárias e Veterinárias, UNESP, Campus de Jaboticabal, 14884-900, Jaboticabal, SP, Brazil; 2 Programa de Pós-Graduação em Genética e Melhoramento Animal, Faculdade de Ciências Agrárias e Veterinárias, UNESP, Campus de Jaboticabal, 14884-900, Jaboticabal, SP, Brazil

**Keywords:** chromosome banding, centric fusion, B chromosomes, multiple sex chromosome system

## Abstract

The Amazonian brown brocket *Mazama nemorivaga* (Cuvier, 1817) is a small to medium-sized deer from the Amazon rainforest and ecotones. The first karyotype described was 2n=67 to 69 + 2-7 B and FN= 69-72, in which all chromosomes were acrocentric and the X chromosome was the only submetacentric chromosome. However, important aspects of the species chromosome evolution were not resolved because of the lack of information on chromosome banding. The G-banding pattern of *Mazama nemorivaga* karyotype showed the presence of an XX/XY_1_Y_2_ sex chromosome system as a product of an X-autosome tandem fusion, which results in a basic 2n=68, FN=70 in females and 2n= 69, FN=70 in males. The fact that this karyotype only differs from that of *Capreolus capreolus pygargus* (Pallas, 1771; 2n=70, FN=72+B) by X-autosome tandem fusion may corroborate the basal condition of *Mazama nemorivaga* and its proximity to the ancestral karyotype of the American Odocoileini. A derived karyotype 2n=67, XY_1_Y_2_, FN=70 + 3B from the Brazilian state of Mato Grosso (the western Amazon) may be evidence of differentiation between western and eastern populations.

## Introduction

The Amazonian brown brocket *Mazama nemorivaga* (Cuvier, 1817) is a small to medium-sized deer that possesses a large muzzle, small ears, protruding eyes, small and spike-like antlers in males and a uniform dark brown coat. This species occurs mainly in the Amazon rainforest and ecotones of Brazil, French Guiana, Surinam, Guyana, Venezuela, Colombia, Ecuador, Peru, and most likely in Bolivia. Although classified as a species of Least Concern by the Red List of the International Union for Conservation of Nature (IUCN) because of its wide distribution, the species would be seriously affected by the effects of deforestation (Rossi et al. 2010). This species belongs to the Rangiferini tribe, which was recently included within the subfamily Capreolinae (González et al. 2010). It was once classified as a subspecies of the gray brocket *Mazama gouazoubira superciliaris* (Cabrera, 1961), but recent molecular analyses demonstrated that *Mazama nemorivaga* is a clearly differentiated species, located basally in the exclusively South American clade of Odocoileinae that also groups together *Blastocerus* (Illiger, 1815),* Ozotoceros* (Linnaeus, 1758),* Hippocamelus* (Molina, 1782), and *Pudu* (Molina, 1782), and which includes *Mazama gouazoubira* (Fisher, 1814) (Duarte et al. 2008). Cytogenetics also support this finding, since the karyotype of *Mazama nemorivaga* presents as 2n=67-69 + 2-7 B and FN=69-72, with a submetacentric X, a very different karyotype from that of *Mazama gouazoubira*: 2n=70+0-3B and FN=70 with an acrocentric X (Rossi et al. 2010). However, the lack of studies on chromosome banding patterns in this species makes it difficult to resolve important aspects of its chromosome evolution. The present study provides much of this information through the analysis of G, C and NOR banding patterns. It also brought new insights for the understanding of the complex chromosome evolution of South American deer.

## Material and methods

**Figure 1.**
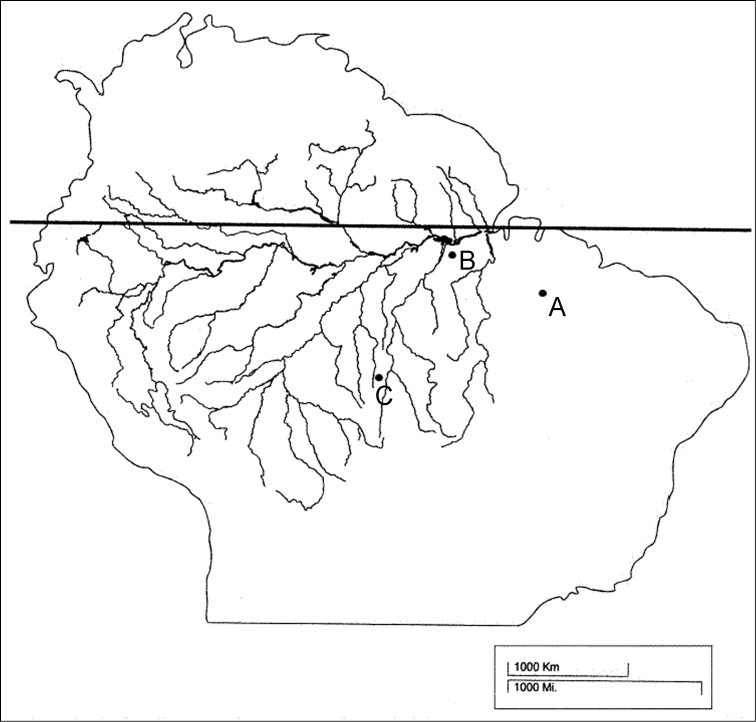
Origin of analyzed animals of the species *Mazama nemorivaga*, where A = animals from Imperatriz, MA (T265), B = animals from Santarém, PA (T261, T262, T263, T264, T266), C = animals from Juina, MT (T275, T295). Modified version of the map by Colinvaux et al. 2000.

Seven wild-caught specimens of *Mazama nemorivaga* were analyzed: one male and four females from the city of Santarém (Pará State, Brazil), one male from the city of Imperatriz (Maranhão State, Brazil), which lies within the eastern Amazon, and one male from the city of Juína (Mato Grosso State, Brazil), which lies within the western Amazon (Figure 1). The males from Imperatriz and Juína and one female are currently being kept in the captive breeding facilities of the “Deer Research and Conservation Center” (NUPECCE – Núcleo de Pesquisa e Conservação de Cervídeos, at São Paulo State University’s, Jaboticabal campus); the other four animals died before the analysis and the cell cultures were prepared from frozen skin samples. The sampling was not homogeneous throughout the *Mazama nemorivaga* distribution area due to the difficulty in capturing free-living animals and the small number of captive specimens in Brazil.

Metaphase chromosomes were obtained from lymphocyte (Moorhead et al. 1960) and fibroblasts (Verma and Babu 1995) cultures using peripheral blood and skin fragments, respectively. The chromosomes were studied using G-banding (Seabright 1971), C-banding (Sumner 1972), and Ag-NOR staining (Howell and Black 1980). Approximately 40 metaphases from each specimen were analyzed in order to determine the diploid number (2n) and the fundamental number (FN). The chromosomes were classified following Abril and Duarte (2008) as metacentric, submetacentric or acrocentric according to their arm ratio, and were then organized into groups according to their relative lengths (RL): A (biarmed chromosomes with RL > 2.5%), C (biarmed chromosomes with RL < 2.5%), D (acrocentric chromosomes with RL < 3.0%), E (acrocentric chromosomes with RL > 3.0%) and B (microchromosomes or extranumerary chromosomes with RL > 1.0%). B chromosomes were not considered in the calculation of the diploid or fundamental numbers because there was intraindividual variation (Abril and Duarte 2008).

**Figure 2.**
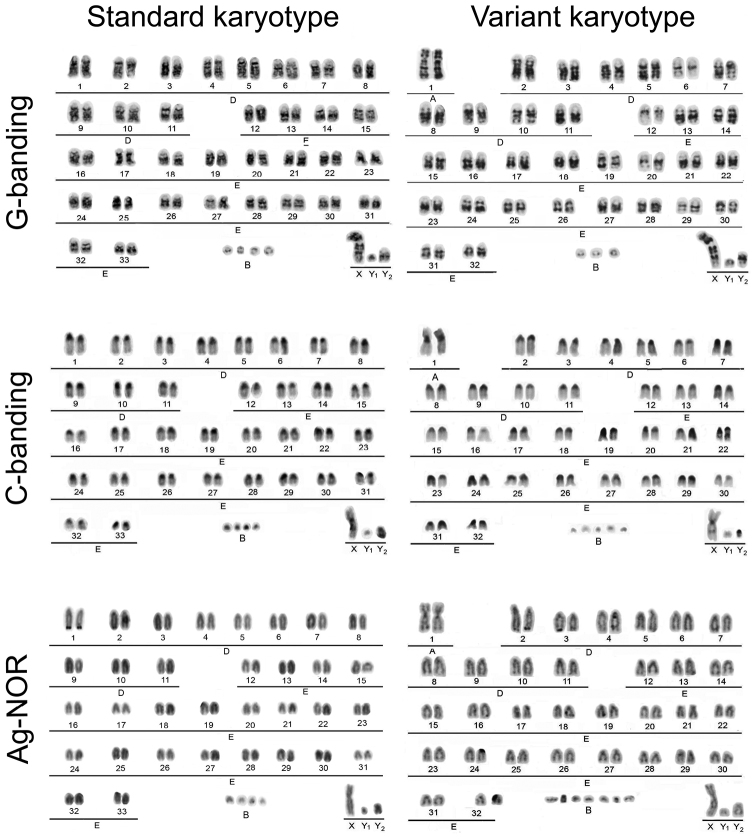
Standard and variant karyotypes of two *Mazama nemorivaga* males after G- and C-banding and Ag-NOR staining.

## Results and discussion

The karyotypes of six animals from the eastern Amazon were found to be standard karyotypes with 2n=68/69 + 2-7B XX/ XY_1_Y_2_ FN=70, composed of 66 acrocentric autosomes from the E group, with the X chromosome being a medium-sized submetacentric chromosome and the males presenting one small metacentric Y_1_ chromosome and one small acrocentric Y_2_ chromosome. The male from the western Amazon had a variant karyotype with 2n=67 + 7B XY_1_Y_2_ FN=70, composed of one pair of large submetacentrics from the A group and 62 acrocentric autosomes from the E group; the sex chromosomes were the same as in the males from the eastern Amazon. The large submetacentric pair in this specimen showed homology with the 4q and 32p of the standard karyotype. The X chromosome showed an interstitial C-band in the middle of the q arm, and its distal half was homologous to the Y_2_ chromosome, which confirm X-autosome tandem fusion. All chromosomes except the Y_1_ chromosome presented extended pericentromeric C-bands. The NOR regions were seen in autosomes as satellites that were distal to the telomeres of the q arms of the two larger acrocentrics (first and second pairs). Additionally, a number of very small supernumerary or B chromosomes, varying from two to seven chromosomes, were present in all of the animals.

The results showed a basic karyotype of 2n=68/69 + 2-6B FN=70, with a sex chromosome system of the XX/XY_1_Y_2_ type for *Mazama nemorivaga*. This kind of sex chromosome system has been widely described for several deer species of the subfamily Muntiacini (Fontana and Rubini 1990), but it is rare in the subfamily Capreoleinae; it has been reported only in the red brocket *Mazama americana* from Brazil (Sarria-Perea 2004, Abril et al. 2010). This rearrangement might has been achieved by both species independently, since they belong to two phylogenetically distant clades (Duarte et al. 2008, Gonzalez et al. 2010).

When comparing the karyotype of *Mazama nemorivaga* to the other species of the subfamily Capreoleinae, its standard karyotype seems to be more similar to that of the Eurasian roe deer species *Capreolus capreolus* (Linnaeus, 1758; with 2n=70, FN=72) and *Capreolus capreolus pygargus* (with 2n=70+10B, FN=72) than to that of other phylogenetically closer South American species such as *Hippocamelus bisulcus* (Molina, 1782) (Vila et al. 2010) and *Pudu puda* (Molina, 1782), which both possess a 2n=70, FN=74 karyotype (Fontana and Rubini 1990). The karyotype of *Mazama nemorivaga* differs from the roe deer karyotypes only by X-autosome tandem fusion and this particular sex chromosome system can be considered as an autapomorphic feature, as the product of an independent process of chromosome evolution. This proximity to the karyotype of the roe deer agrees with molecular phylogenetics that arranges *Mazama nemorivaga* as a basal species onto the South American Rangiferini clade (Duarte et al. 2008, Gonzalez et al. 2010). The proximity may also suggest that the ancestral karyotype of the New World Rangiferini is likely to be 2n=70, FN=72 rather than the 2n=70, FN=70 karyotype that has previously been reported in the literature (Fontana and Rubini 1990).

The single animal from the western Amazon analyzed here had a different karyotype, which was composed of 64 autosomes, with one pair from the A group. According to its G-banding patterns, this new submetacentric pair was the product of the Robertsonian translocation between the chromosomes 4 and 32 from standard karyotype. The fixation of this kind of rearrangement in some local population increases a probability of reproductive isolation of this population. Such a process has indeed been observed in a sympatric brocket deer species *Mazama americana* (Abril et al. 2010).

These results seem to indicate that *Mazama nemorivaga* has a karyotype similar to the ancient one, but it followed an independent and complex chromosomal evolutionary pathway. The existence of polymorphic karyotypes likely indicate some degree of population differentiation and this can be found in other neotropical brocket deer species (Abril and Duarte 2008; Abril et al. 2010). Despite the small number of analyzed animals in this study, these are the first results concerning the karyotypic status of the *Mazama nemorivaga* in Brazil, and they are fundamental for a new review of chromosomal evolution in Cervidae.
